# Present and Future of EGFR Inhibitors for Head and Neck Squamous Cell Cancer

**DOI:** 10.1155/2012/986725

**Published:** 2012-03-26

**Authors:** Yuh Baba, Masato Fujii, Yutaka Tokumaru, Yasumasa Kato

**Affiliations:** ^1^Department of Otorhinolaryngology and Head and Neck Surgery, Ohtawara Red Cross Hospital, 2-7-3, Sumiyoshi-cho, Ohtawara City, Tochigi 324-8686, Japan; ^2^National Institute of Sensory Organs, National Tokyo Medical Center, 2-5-1, Higashigaoka, Meguro, Tokyo 152-8902, Japan; ^3^Department of Oral Function and Molecular Biology, Ohu University, 31-1, Mitsumido, Tomita-machi, Koriyama City, Fukushima 963-8611, Japan

## Abstract

Although EGFR is expressed at high levels in head and neck squamous cell carcinomas (HNSCCs) and mutations are extremely rare, monotherapy with EGFR inhibitors has shown limited success. The PI3kinase/Akt pathway is responsible for cellular survival, and inhibition of phosphatidylinositol (PI) synthesis has antiproliferative, anti-invasive, and antiangiogenesis effects on HNSCC. Molecular crosstalk has been observed between EGFR and IGF1R signaling through the PI3kinase/Akt pathway in HNSCC, as has molecular crosstalk between the NF**κ**B and STAT3 signaling pathways. Therefore, the combination of an EGFR antagonist with an agent that inhibits the activation of both Akt and NF**κ**B may overcome resistance to EGFR antagonists in HNSCC.

## 1. Introduction

 head and neck squamous cell carcinoma (HNSCC) is the sixth most common neoplasm worldwide, with approximately 600,000 patients newly diagnosed each year [1]. Over the past 30 years, patients with recurrent and/or metastatic HNSCC have had a poor prognosis [2, 3]. More than 50% of newly diagnosed patients do not achieve complete remission, and approximately 10% relapse with metastasis to distant organs [4]. Therefore, research focused on gaining a better understanding of this disease and on the development of novel treatment strategies is required.

Epidermal growth factor receptor (EGFR), a ubiquitously expressed transmembrane glycoprotein belonging to the ErbB/HER family of receptor tyrosine kinases (TK), is composed of an extracellular ligand-binding domain, a hydrophobic transmembrane segment, and an intracellular TK domain. Upon ligand binding to EGFR, the latter undergoes a conformational change that promotes homo- or heterodimerization with other members of the ErbB/HER family of receptors, followed by autophosphorylation and activation of the TK domain [5]. Activation of EGFR leads to activation of intracellular signaling pathways that regulate cell proliferation, invasion, angiogenesis, and metastasis.

EGFR is expressed at high levels in the majority of epithelial malignancies including HNSCC [6]. Elevated expression of EGFR in HNSCC correlates with poor prognosis, and EGFR has been a target of anticancer treatments due to its critical roles in cell survival and proliferation [7]. Among the tyrosine kinase inhibitors targeting EGFR that have been approved by the US FDA are gefitinib, erlotinib, and lapatinib [8]. These molecules are reversible competitors, competing with adenosine triphosphate (ATP) for the tyrosine kinase binding domain of EGFR. Inhibition of receptor activation inhibits downstream signaling pathways, resulting in decreased cell proliferation and survival. EGFR signaling activates a number of downstream effectors including the phosphatidylinositol-3-kinase (PI3K)/Akt pathway.

## 2. Rare EGFR Mutations in HNSCC

Somatic mutations in the TK domain of the EGFR gene, including in-frame deletions in exon 19 and the point mutations L858, G719X, and L861Q, are associated with increased sensitivity to EGFR tyrosine kinase inhibitors (TKIs) and are present in 10~30% of patients with nonsmall cell lung carcinoma (NSCLC), depending on ethnic origin. These mutant EGFRs selectively activate signal transduction and activator of transcription (STAT) and Akt signaling pathways, which promote cell survival.

 However, they have no effect on extracellular signal-regulated kinase (ERK) signaling, which induces cell proliferation. Furthermore, mutant EGFRs selectively transduce survival signals, and inhibition of these signals may contribute to the efficacy of TKIs used to treat NSCLC [9]. However, molecular analysis of HNSCC tumor samples has not revealed the same spectrum of mutations [10–12].

One important resistance mutation in EGFR is the T790M missense mutation in the kinase domain, which may contribute to TKI resistance in NSCLCs possessing the L858R point mutation [13]. Using the Cycleave PCR method, however, we failed to detect the T790M mutation in 86 HNSCC tumor samples [14].

## 3. Resistance to EGFR TKIs

EGFR TKIs have had limited results in patients with HNSCC. For example, a phase II trial of gefitinib in patients with recurrent or metastatic HNSCC showed an overall response rate of 11% [15]. Similarly, a study of erlotinib in patients with recurrent and/or metastatic HNSCC showed a response rate of 4% [16]. Four mechanisms have been proposed to explain tumor resistance to EGFR TKIs.

### 3.1. Ras Mutations

*K*-*ras *mutations may cause tumor insensitivity to EGFR TKIs. Activating *K*-*ras* mutations may activate the Ras/mitogen-activated protein kinase (MAPK) pathway independent of EGFR, thus inducing resistance to EGFR TKIs [17]. *H*-*ras* mutations are more common than *K*-*ras* mutations in HNSCC and may play an important role in tumor resistance to EGFR-targeted therapies [18].

### 3.2. Epithelial-Mesenchymal Transition (EMT)

EMT results in changes in cell morphology and motility and is indicated by increased expression of vimentin and claudins 4 and 7 and by decreased expression of E-cadherin. EMT has been associated with gefitinib resistance in HNSCC [19].

### 3.3. Upregulation of Cyclin D1

Upregulation of cyclin D1 in HNSCC cell lines has been specifically associated with resistance to gefitinib. Upregulation of cyclin D1 results in the activation of cyclin D1-cyclin-dependent kinase 4 (CDK4), which hyperphosphorylates retinoblastoma protein (pRb) [20].

### 3.4. PI3Kinase/Akt Signaling as a Dominant Pathway

Increased expression of cortactin, a protein that increases the formation of actin networks critical to cell motility and receptor-mediated endocytosis, has been associated with gefitinib resistance and increased metastasis in HNSCC [21].

Akt has been implicated in EMT by integrin-linked kinase (ILK). The PI3K/Akt pathway not only regulates the transcriptional activity of cyclin D1 but also increases its accumulation by inactivating glycogen synthase kinase-3 (GSK3), an enzyme that targets cyclin D1 for proteasomal degradation. Cortactin is thought to promote cancer cell proliferation by activating Akt [21], suggesting that factors related to resistance to EGFR TKIs are associated with the PI3K/Akt pathway.

## 4. PI3K/Akt Pathway

In this section, we will explain the activation of the PI3K/AKT pathway, its downstream effectors, and the rationale for targeting this pathway in HNSCC.

### 4.1. Activation of the PI3K/Akt Pathway

Signaling through the PI3K/Akt pathway can be initiated by several mechanisms. Once activated, this pathway can be propagated to various substrates, including mTOR, a master regulator of protein translation. The PI3K/Akt pathway is initially activated at the cell membrane, where the signal for activation is propagated through class IA PI3K. Activation of PI3K can occur through tyrosine kinase growth factor receptors such as EGFR and insulin-like growth factor-1 receptor (IGF-1R), cell adhesion molecules such as integrins, G-protein-coupled receptors (GPCRSs), and oncogenes such as Ras. PI3K catalyzes the phosphorylation of the D3 position on phosphoinositides, generating the biologically active moieties phosphatidylinositol-3,4,5-triphosphate (PI(3,4,5)P3) and phosphatidylinositol-3,4-bisphosphate (PI(3,4)P2). PI(3,4,5)P3 binds to the pleckstrin homology (PH) domains of 3′-phosphoinositide-dependent kinase 1 (PDK-1) and Akt, resulting in the translocation of these proteins to the cell membrane, where they are subsequently activated. The tumor suppressor phosphatase and tensin homolog deleted on chromosome ten (PTEN) antagonizes PI3kinase by dephosphorylating PI(3,4,5)P3 and (PI(3,4)P2), thereby preventing the activation of Akt and PDK-1. Akt exists as three structurally similar isoforms, Akt1, Akt2, and Akt3, which are expressed in most tissues. Activation of Akt1 occurs through two crucial phosphorylation events. The first, catalyzed by PDK-1, occurs at T308 in the catalytic domain of Akt1. Full activation requires a subsequent phosphorylation at S473 in the hydrophobic motif of Akt1, a reaction mediated by several kinases, including PDK-1, ILK, Akt itself, DNA-dependent protein kinase, and mTOR; phosphorylation of homologous residues in Akt2 and Akt3 occurs by the same mechanism. Phosphorylation of Akt at S473 is controlled by a recently described phosphatase, PH domain leucine-rich repeat protein phosphatase (PHLPP), which has two isoforms that preferentially decrease the activation of specific Akt isoforms [22]. Amplification of Akt1 has been described in human gastric adenocarcinomas, and amplification of Akt2 has been described in ovarian, breast, and pancreatic carcinomas [23, 24]. Akt mutations are rare, but somatic mutations have been reported in the PH domain of Akt1 in a small percentage of human breast, ovarian, and colorectal cancers [25]. 

### 4.2. Downstream Substrates of Activated Akt

Akt recognizes and phosphorylates the consensus sequence RXRXX (S/T) when it is surrounded by hydrophobic residues. Since this sequence is present in many proteins, Akt has many substrates, many of which control key cellular processes such as apoptosis, cell cycle progression, transcription, and translation. For example, Akt phosphorylates proteins in the FoxO subfamily of forkhead family transcription factors, inhibiting the transcription of several proapoptotic genes including Fas-L, IGF binding protein1 (IGFBP1), and Bim. In addition, Akt can directly regulate apoptosis by phosphorylating and inactivating proapoptotic proteins such as BAD, which controls the release of cytochrome c from mitochondria, and apoptosis signal-regulating kinase-1 (ASK1), a mitogen-activated protein kinase kinase involved in stress- and cytokine-induced cell death. In contrast, Akt can phosphorylate IKK, which indirectly increases the activity of nuclear factor kappa B (NF-*κ*B) and stimulates the transcription of prosurvival genes. Cell cycle progression can also be affected by Akt; inhibitory phosphorylation of the cyclin-dependent kinase inhibitors p21 and p27, and inhibition of GSK3 *β* by Akt, stimulates cell cycle progression by stabilizing cyclin D1 expression. A novel prosurvival Akt substrate, proline-rich Akt substrate of 40 kDa (PRAS40), has been described recently [26]. Phosphorylation of PRAS40 by Akt attenuates its ability to inhibit mTORC1 kinase activity. PRAS40 may be a specific substrate of Akt3 [27]. Therefore, Akt inhibition may have pleiotropic effects on cancer cells that contribute to an antitumor response. The most studied downstream substrate of Akt is the serine/threonine kinase mammalian target of rapamycin (mTOR). Akt can directly phosphorylate and activate mTOR, as well as indirectly activating it by phosphorylating and inactivate tuberous sclerosis complex 2 (TSC2), also called tuberin, which normally inhibits mTOR through the GTP binding protein Ras homolog enriched in brain (Rheb) [28]. When TSC2 is inactivated by phosphorylation, the GTPase Rheb is maintained in its GTP-bound state, allowing increased activation of mTOR [29]. mTOR exists in two complexes: the TORC1 complex, in which mTOR is bound to Raptor; and the TORC2 complex, in which mTOR is bound to Rictor. In the TORC1 complex, mTOR signals its downstream effectors, S6 kinase/ribosomal protein and 4EBP-1/eIF-4E, to control protein translation [29]. mTOR is generally considered a downstream substrate of Akt, but it can phosphorylate Akt when bound to Rictor in TORC2 complexes [30], resulting in positive feedback in the pathway. In addition, the downstream mTOR effector S6 kinase-1 (S6K1) can regulate this pathway by catalyzing the inhibitory phosphorylation of insulin receptor substrate (IRS) proteins. This prevents IRS proteins from activating PI3kinase, thereby inhibiting the activation of Akt [31].

### 4.3. Rationale for Targeting the PI3K/Akt Pathway

In addition to preclinical studies, clinical observations support the targeting of the PI3K/Akt/mTOR pathway in human cancer [32]. Immunohistochemical studies using antibodies that recognize Akt phosphorylated at S473 have demonstrated that activated Akt is detectable in cancers including head and neck cancers [33]. Moreover, using antibodies against S473 and T308, two sites of Akt phosphorylation, Akt activation was shown to be selective for NSCLC versus normal tissue, and phosphorylation of Akt at both sites was shown to be a better predictor of poor prognosis in NSCLC than phosphorylation at S473 alone [34]. In addition, amplification of Akt isoforms has been observed in some cancers, albeit at a lower frequency. Another frequent genetic event occurring in human cancer is loss of function of the tumor suppressor PTEN, which normally suppresses activation of the PI3K/Akt/mTOR pathway by functioning as a lipid phosphatase. Loss of PTEN function in cancer can occur through mutation, deletion, or epigenetic silencing. In tumor types where PTEN mutations are rare, such as lung cancer, epigenetic silencing can occur [35]. Mutation, deletion, or epigenetic silencing of PTEN has been shown to correlate with poor prognosis and reduced survival in patients with various types of cancer [36], with loss of PTEN being a common mechanism for activation of the PI3K/Akt/mTOR pathway and poor prognosis. Activation of PI3K has been described in human tumors that may result from the amplification, overexpression, or mutation of the p110 catalytic or p85 regulatory subunit. Amplification of the 3q26 chromosomal region, which contains the *PI3KCA *gene that encodes the p110*α* catalytic subunit of PI3K, has been observed in 40% of ovarian and 50% of cervical carcinomas [37, 38]. Somatic mutations of this gene have been detected in several cancer types, with mutant PI3K having increased kinase activity relative to wild type [39]. Mutations in the regulatory p85 subunit have also been detected. Any of the alterations in individual components of the PI3 kinase/Akt pathway would result in its activation, and activation of this pathway has been reported to be among the most frequent molecular alterations in tumors [39].

## 5. Inhibition of PI Synthesis in HNSCC

PIP2, a substrate of PI3K, may be synthesized from PI by the PI4 and PI5 kinases. Therefore, inhibition of PI metabolic pathway may be an important antitumor strategy. Our laboratory has investigated three potential mechanisms by which inhibition of PI synthesis inhibition may affect patients with HNSCC. These are antiproliferation, inhibition of matrix metalloproteinase (MMP) production/activity, and antiangiogenesis.

### 5.1. Antiproliferation

An imbalance between G1 cyclin and cyclin-dependent kinase (CDK) inhibitors (CKIs) has been reported to contribute to tumorigenesis and tumor progression. Cyclin D1/PRAD1 acts as a positive regulator of the cell cycle by phosphorylating pRB (Rb protein) and by forming a cyclin D1-CDK4 complex. Upon hyperphosphorylation by CDKs, pRB releases E2F, a factor necessary for activating a gene expression network that regulates entry and progression through S phase.

CKIs can be classified into two groups: members of the Ink4 family (p15, p16, p18, and p19), which are incorporated into cyclin D/CDK4 and cyclin D/CDK6; and members of the cip/kip family (p21, p27, and p57), which are incorporated into cyclin D/CDK4 and cyclin E/CDK2. Overexpression of cyclin D1 in HNSCC is an important prognostic marker, predicting sensitivity to chemotherapy and radiotherapy. Furthermore, imbalances between cyclin D1 and its inhibitors (p16 and p27) may be critical in the development of HNSCC. Strategies to block cyclin D1 function have been studied extensively. For example, introduction of an antisense cyclin D1 expression vector into cells reduced their growth rate *in vitro* and decreased tjeor tumorigenicity in athymic nude mice [40]. We previously reported that inhibition of PI synthesis caused G1 arrest of HNSCC, accompanied by decreased levels of cyclin D1, cyclin E, and phosphorylated pRB [41].

### 5.2. Inhibition of MMP Production/Activity

Tumor metastasis is a complex multistep process, including growth at the primary site, entry into the circulation (intravasation), adhesion to basement membranes (BMs) of target organs, extravasation, and growth at secondary sites. The intravasation and extravasation processes involve degradation of the BM by proteinases, normally MMPs. MMP-9/gelatinase B and MMP-2/gelatinase A are specific for type IV collagen, which acts as the backbone of BM, and therefore probably play a major role in degrading the BM. In HNSCC, MMP-2 and MMP-9 are associated with metastatic potential. Therefore, MMPs are attractive targets for therapy and many drugs have been developed to prevent their extracellular matrix-degrading activities during metastasis and angiogenesis. We previously demonstrated that inhibition of PI synthesis affects the production of MMP-2 and MMP-9 in HNSCC cell lines [42].

### 5.3. Antiangiogenesis

Angiogenesis, the formation of new blood vessels from preexisting capillaries or incorporating bone marrow-derived endothelial precursor cells into growing vessels, is associated with the malignant phenotype of cancer. Angiogenesis is also involved in other diseases, including diabetic retinopathy, age-related macular degeneration, rheumatoid arthritis, psoriasis, atherosclerosis, and restenosis [43]. Since tumor vascularity has been associated with tumor aggressiveness in many tumor types, including HNSCC, determining microvessel density in tumor tissues may be useful in estimating patient prognosis. Inhibition of angiogenesis can repress the growth rate of tumor cells and lead to cell death resulting from reduced nutrition and oxygen supply to the tumor. Upon binding to its receptor Flk-1/KDR, vascular endothelial growth factor (VEGF), which plays a major role in many angiogenic processes, stimulates endothelial cell (EC) proliferation through the phospholipase C*γ*-protein kinase extracellular signal-regulated kinase (C-ERK) pathway, but not via Ras [44]. VEGF also stimulates EC migration through p38 mitogen-activated kinase (MAPK) independently of ERK [45]. Therefore, these two major MAPK pathways may be therapeutic targets for reduction of angiogenesis in HNSCC.

Most clinical trials of antiangiogenic agents have been performed in patients with advanced disease who had become resistant to conventional therapies. Phase III trials of these agents have compared the efficacy of standard chemotherapy alone and in combination with an experimental angiogenesis inhibitor [46]. The results of some studies were negative or unclear, but several recent clinical trials have demonstrated a significant clinical benefit of VEGF inhibition [47]. Sunitinib, a tyrosine kinase inhibitor of the Flk-1/KDR receptor (VEGF receptor), and bevacizumab, a monoclonal antibody against VEGF, have been approved by the FDA [47]. Furthermore, we have demonstrated that the inhibition of PI abrogated VEGF stimulation of the growth and migration of human umbilical vein ECs through the ERK-cyclin D1 and p38 pathways, respectively [48]. Since increased expression of PI synthase is an early event in HNSCC [49], inhibition of PI synthesis may be a potent therapeutic strategy in patients with HNSCC [50].

## 6. Crosstalk between the IGF1R and EGFR Pathways

### 6.1. Pathway Switching between EGFR and IGF1R

Growth factor switching from one pathway to another may be an adaptive mechanism, induced by blocking the dominant growth factor receptor pathway. Blockade of EGFR signaling in DU145 and PC-3 human prostate cancer cells has found to enhance the growth promoting effects of the peptide growth factor ligands basic fibroblast growth factor (bFGF) and IGF-1, respectively [51]. More recently, the EGFR-selective tyrosine kinase inhibitor gefitinib has been shown to inhibit the growth of EGFR-positive MCF-7-derived tamoxifen-resistant breast cancer cells, an effect that can be abrogated by exposing the cells to non-EGF ligands such as heregulin-*β* and IGF-II [52]. The reversal of the antitumor effects of gefitinib by IGF-II, acting through the IGF-1R, is accompanied by reactivation of the previously reduced activity of Akt and extracellular-regulated kinase (ERK), with ERK signaling contributing to the reestablishment of tumor cell growth. Therefore, in the presence of a dominant growth pathway, cancer cells are capable of responding to other growth factors, compromising the antitumor activity of agents designed specifically to inhibit EGFR. Importantly, blockade of EGFR signaling frequently results in switching to the IGF-1R pathway, a common mechanism used to promote resistance to anti-EGFR treatment [53]. For example, gefitinib initially inhibited the growth of the EGFR-positive DU145 prostate cancer cell line and of MCF-7-derived tamoxifen- and fulvestrant-resistant breast cancer cell lines, but chronic exposure to gefitinib resulted in the development of gefitinib-resistant variant sublines, all of which showed upregulation of multiple IGF-1R signaling components when compared with their parental cell lines [54]. This resulted in increased production and elevated expression of the IGF-1R, ligand IGF-II, increased activity of IGF-1R and increased levels of Akt activity. In addition, although the A549 lung cancer cell line is partially sensitive to gefitinib, chronic exposure resulted in a resistant variant with increased activity of elements of the IGF-1R pathway. The importance of IGF-1R signaling in cell lines with acquired gefitinib resistance was supported by their increased dependency on IGF-1R signaling and their greater sensitivity to growth inhibition by IGF-1R-selective TKIs [54]. Therefore, the dominance of the EGFR pathway in parental cells was replaced by an increased use of the IGF-1R pathway in gefitinib resistant cells.

Growth factor pathway switching not only may result from changes occurring during the development of acquired resistance but also, critically, may occur rapidly and modulate initial sensitivity to EGFR-blockade, resulting in *de novo *or intrinsic resistance to anti-EGFR agents such as gefitinib. Indeed, although the EGFR and IGF-1R pathways are classically regarded as separate entities, the overlapping of downstream signal transduction molecules indicates that these receptors can affect each other's signaling abilities, although the precise mechanisms involved in this crosstalk have not been fully elucidated. For example, gefitinib only partially blocks EGFR activity in A549 lung cancer cells, accompanied by a dramatic increase in the activity but not the expression of IGF-1R. Moreover, in these cells IGF-1R can transphosphorylate EGFR, maintaining EGFR activity in the presence of gefitinib. Therefore, by enhancing IGF-1R activity, gefitinib limits its own efficacy in these cells. Interestingly, it was observed that, in *de novo* gefitinib-resistant LoVo colorectal cancer cells, which are defective in their ability to produce mature IGF-1R and predominantly express insulin receptor-isoform A (InsR-A), a close family member of the IGF-1R, gefitinib enhances insulin receptor activity and levels of downstream activated Akt [55]. Furthermore, InsR can modulate and maintain EGFR phosphorylation in these cells. Such rapid and dynamic interplay between EGFR and IGF-1R or InsR may play an important role in limiting the antitumor activity of gefitinib; partial and *de novo *resistance to this inhibitor has been demonstrated in A549 and LoVo cells, respectively.

### 6.2. Cotargeting the EGFR and IGF1R in HNSCC

Treatment of HNSCC cells and xenografts with the combination of antibodies to IGF-1R and EGFR was more effective than either agent alone at reducing cancer cell growth [56], suggesting a potential benefit in the use of combined anti-tyrosine kinase receptor directed therapies to treat HNSCC. Similarly, the use of small molecules targeting these two pathways suppressed the growth of HNSCC cells [57], as did the combination of cetuximab with a PI3K inhibitor HNSCC [58].

## 7. Crosstalk between the NF*κ*B and STAT3 Signaling Pathways

Although EGFR activation has been found to lead to the rapid phosphorylation of STAT3 on tyrosine 705 and the subsequent activation of STAT3-dependent gene expression, STAT3 tyrosine phosphorylation and the formation of active STAT3 DNA-binding complexes were insensitive to EGFR inhibition in many HNSCC cell lines [59]. Indeed, of a representative panel of 10 HNSCC-derived cell lines, 9 showed increased tyrosine phosphorylation and STAT3 activity, but only 3 showed constitutive activation of EGFR [59]. In searching for the mechanism responsible for the EGFR-independent activation of STAT3 in HNSCC cells, the activation of the gp130 cytokine receptor subunit was found to promote the phosphorylation of STAT3 at tyrosine 705 through the activation of intracellular tyrosine kinases of the JAK family. Surprisingly, gp130 activation was found to be primarily initiated by IL-6, which is secreted by HNSCC cells and binds to the cell surface in an autocrine fashion. These findings suggest that the persistent activation of STAT3 in HNSCC can result from the deregulation of EGFR activity or from the EGF-independent autocrine activation of STAT3 by tumor-secreted cytokines. Furthermore, overexpression of IL-6 in HNSCC cells was found to involve increased transcription from the IL-6 promoter, which is dependent on the presence of an intact NF*κ*B response element located 63 to 75 bp upstream of the IL-6 transcriptional initiation site. Inhibition of NF*κ*B resulted in the marked downregulation of IL-6 mRNA and protein expression, concomitant with the decreased release of other inflammatory cytokines, such as IL-8, IL-10, granulocyte-macrophage colony-stimulating factor (GM-CSF), and granulocyte colony-stimulating factor (G-CSF). Surprisingly, the blockade of NF*κ*B also resulted in the drastic inhibition of constitutive STAT3 activity in HNSCC cells, as reflected by the reduced tyrosine phosphorylation of STAT3. Interestingly, interfering with NF*κ*B function also prevented the autocrine/paracrine activation of STAT3 in HNSCC cells [60]. These findings support the crosstalk between the NF*κ*B and the STAT3 signaling systems. This crosstalk is initiated by the release of IL-6, resulting from the NF*κ*B-dependent activation of the IL-6 promoter, and the subsequent tyrosine phosphorylation of STAT3 by the autocrine/paracrine activation of IL-6 receptors in tumor cells.

## 8. Future Prospects

**Figure 1:**
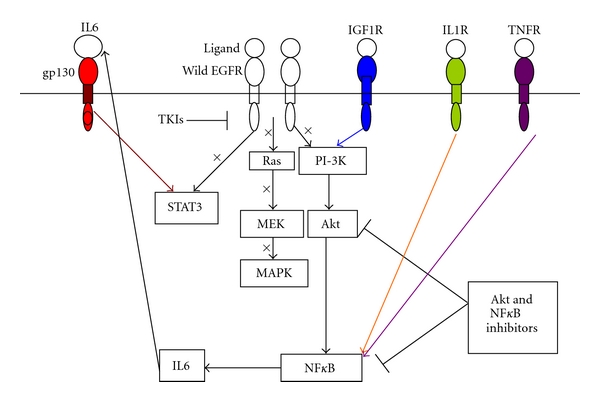
Proposed mechanism for overcoming HNSCC resistance to EGFR antagonists using PI3kinase/Akt/mTOR and NF*κ*B-IL6-STAT3 pathway inhibitors.

Signaling of multiple receptor tyrosine kinases (RTKs) is propagated through Akt. Therefore, simultaneous inhibition of EGFR, as well as pathway components such as Akt or mTOR, could circumvent the feedback activation observed with either approach alone. The most extensive data concerning proximal and distal signaling inhibition has been observed by combining PI3K/Akt/mTOR pathway inhibitors with EGFR antagonists. Several PI3K inhibitors can restore cellular sensitivity to EGFR inhibitors. For example, the selective pI3K inhibitor PX-866 and p110*α* were found to abrogate gefitinib resistance in NSCLC xenografts [61]. Synergistic effects of rapamycin and EGFR TKIs have been observed in several *in vitro* systems, including glioblastoma multiforme, prostate cancer, pancreatic cancer, squamous cell carcinoma, renal cell carcinoma, leukemia, cervical carcinoma, and NSCLC cell lines, as well as in some xenografts [62–67]. The combination of rapamycin and erlotinib showed resensitization and synergistic growth inhibition in cell lines that were previously resistant to erlotinib [64]. Moreover, the combination of rapamycin and the irreversible EGFR TKI, HKI-272, resulted in the significant regression of lung tumors in transgenic mice possessing the secondary resistance mutation T790M [68]. Addition of the dual PI3K/mTOR inhibitor PI-103 to erlotinib was necessary to induce growth arrest of human glioma cell lines with mutant PTEN [69], suggesting that activation of the PI3K/Akt/mTOR pathway by EGFR-independent mechanisms confers resistance to EGFR inhibitors, but that this resistance can be overcome by the addition of pathway inhibitors. Collectively, these findings suggest that the combination of EGFR antagonists and PI3K/Akt pathway inhibitors may be beneficial to patients with tumors resistant to EGFR TKIs. These combinations, however, may be insufficient for the treatment of patients with HNSCC, due to the crosstalk between the NF*κ*B and STAT3 signaling pathways, as described in Section 7, (Figure 1). In these patients, the triple combination of EGFR antagonists with PI3K/Akt/mTOR pathway inhibitors and NF*κ*B-IL6-STAT3 pathway inhibitors may be effective (Figure 1).

## 9. Conclusions

EGFR is expressed at a high level in HNSCC, but EGFR inhibitor monotherapy has had limited success in patients with these tumors. EGFR mutations are extremely rare in HNSCC, whereas inhibition of PI synthesis has antiproliferative, anti-invasive, and antiangiogenesis effects on HNSCCs. The PI3K/Akt pathway is responsible for cellular survival and there is molecular crosstalk between EGFR and IGF1R signaling through PI3K/Akt in HNSCCs. Furthermore, there is molecular crosstalk between the NF*κ*B and STAT3 signaling pathways. Therefore, combination therapy targeting these three signaling pathways, PI3K/Akt, NF*κ*B/STAT3, and EGFR, may provide clinical benefits for patients with HNSCC.
